# Maternal care and birth outcomes among ethnic minority women in Finland

**DOI:** 10.1186/1471-2458-9-84

**Published:** 2009-03-20

**Authors:** Maili Malin, Mika Gissler

**Affiliations:** 1 National Institute for Health and Welfare, Mannerheimintie 166, 00300 Helsinki, Finland; 2 Nordic School of Public Health, Gothenburg, Sweden

## Abstract

**Background:**

Care during pregnancy and labour is of great importance in every culture. Studies show that people of migrant origin have barriers to obtaining accessible and good quality care compared to people in the host society. The aim of this study is to compare the access to and use of maternity services, and their outcomes among ethnic minority women having a singleton birth in Finland.

**Methods:**

The study is based on data from the Finnish Medical Birth Register in 1999–2001 linked with the information of Statistics Finland on woman's country of birth, citizenship and mother tongue. Our study data included 6,532 women of foreign origin (3.9% of all singletons) giving singleton birth in Finland during 1999–2001 (compared to 158,469 Finnish origin singletons).

**Results:**

Most women have migrated during the last fifteen years, mainly from Russia, Baltic countries, Somalia and East Europe. Migrant origin women participated substantially in prenatal care. Interventions performed or needed during pregnancy and childbirth varied between ethnic groups. Women of African and Somali origin had most health problems resulted in the highest perinatal mortality rates. Women from East Europe, the Middle East, North Africa and Somalia had a significant risk of low birth weight and small for gestational age newborns. Most premature newborns were found among women from the Middle East, North Africa and South Asia. Primiparous women from Africa, Somalia and Latin America and Caribbean had most caesarean sections while newborns of Latin American origin had more interventions after birth.

**Conclusion:**

Despite good general coverage of maternal care among migrant origin women, there were clear variations in the type of treatment given to them or needed by them. African origin women had the most health problems during pregnancy and childbirth and the worst perinatal outcomes indicating the urgent need of targeted preventive and special care. These study results do not confirm either healthy migrant effect or epidemiological paradox according to which migrant origin women have considerable good birth outcomes.

## Background

The care given during pregnancy and labour is of great importance in every culture. After migration different cultures meet at childbirth, a very sensitive moment in life. Besides personal encounters, it is a situation in which parturients and their families from different parts of the world meet with western midwives, doctors and overall maternal health care with its institutional culture and practices. Because increasingly ethnically diverse migrant women resettle in industrialised countries, several social, psychological and biological factors need to be considered in caring for them during pregnancy and labour. From previous studies we know that non-western origin migrant women are more often multiparous, have more pregnancy-related risk factors, and have more infectious diseases which further may have adverse health effect to them and their newborns [1-4]. One UK study reported non-white ethnicity to be one of predictors of severe obstetric morbidity [5].

In an ideal situation when studying the access to maternal health care and birth outcomes of ethnic minority women we have to know their health needs and health status during pregnancy, what factors may affect and have affected to their health, what kind of help seeking behaviour they have and how the service system functions for them. Help-seeking behaviour is affected at least by a person's concepts of health and illness, health literacy, know how about the functioning of health system, economic possibilities to seek help, and past experiences of services and care [6,7]. Utilisation or accessibility of care refers to the volume of service usage and service given, the site of utilisation and access and the type of services used and having access [7]. Beside many individual/patient-related factors, access to care can be affected by a number of barriers related to ward level, supply and provision of care, such as a lack of necessary professionals and facilities, long distance to travel for the necessary care procedures [8,9], communication problems between the care giver and patient due to the missing language skills or improper attitudes, or when care givers' referral practices differ depending on the social characteristics of the patients [10,11].

Recently, there has been an explosion of empirical evidence of ethnic disparities in medical care in regard to clinical appropriateness, to treatment site and to other clinical factors [12-19]. These ethnic disparities in service delivery are not unique to medical care, since there are similar documentations in the fields of justice, child welfare [20], education, labour and housing [12]. Structural, indirect and direct discrimination is recognised to cause partly these ethnic disparities in the amount and the content of public services as well as in the living conditions and opportunities. It has been indicated that when patients' non-White ethnicity is combined with a lower social class position, the care providers' prejudices and care giving is in increased risk of being discriminatory. [21-24]

Ethnic disparities in maternity care and health outcomes seem to be persisting in those societies where ethnic minorities have existed for centuries. In the US, the perinatal outcomes (low birth weight, preterm birth and mortality) for African American newborns are still much worse than for White Americans [25]. African Americans are also less likely to receive prenatal preventive care advice regarding smoking cessation, alcohol use and breastfeeding [26]. Young Blacks and Hispanics have notably higher risks of adverse birth outcomes indicating less access to prenatal care of reasonable quality [27]. In terms of the content of care it has been claimed that (White) doctors behave less affectively when interacting with ethnic minority patients compared to White non-migrants, and the ethnic minority patients have been stated to be less assertive during medical encounters [28].

In multicultural countries, e.g. Canada [7] and the UK [29], migrant origin people use health care services less than they need, terminate their care earlier than is advantageous for the cure, and receive lower quality care than others. In a comparative European study [30], women who did not use maternal care as recommended were identified to be of foreign origin, teenagers, multiparous, single and with an unplanned pregnancy. Furthermore, they were more often less educated and without regular income. In the Netherlands [31], all non-Dutch ethnic groups were significantly later in starting antenatal care during their pregnancy compared with the ethnic Dutch group. Ethnic disparities in maternal health care have been found also in Sweden and Norway [3,4,32-36], in Italy [37] and in Switzerland [38].

### Migrants in Finland

Finland is situated on the eastern border of Europe next to Russia creating one of the highest gaps in the standard of living. For these reasons most of the migrants in Finland are from the neighbouring areas such as Russia and the Baltic countries due to work, marriage and as returnees (having Finnish ancestors). Finland became a multicultural society only after the collapse of the Soviet Union and after the remarkable increase in non-Western refugees during the past decades. Thus, there is a small but growing ethnic minority population (determined by the country of origin) in Finland which at the moment constitutes 4% of the whole population and due to their concentration to the south 9% of the population in the City of Helsinki [39]. Migrant origin women form very heterogeneous population from those who migrate from EU-countries voluntarily to those women who come from outside western world having left their countries by force (e.g., war, persecution). Many of those latter are separated from their families, have limited language skills, and are many times visible minorities. Thus, the health needs of these ethnic minority women vary considerable.

In this study a person with migrant origin or being ethnic minority is a person who has permanent residency in Finland and whose mother language is other than Finnish or Swedish, and her country of birth is other than Finland. Thus, a migrant origin woman could have come to the country for example as a refugee, an asylum seeker, a worker, a student, through a resettlement program or family unification, or as a result of marriage. Most of those who has migrated to Finland are young people living their productive and reproductive years [40,41]. Half of the migrants to Finland are female although there are some culture-specific gender differences in their numbers, for example most Russians, Thais and Filipinos are women married with Finnish men [40].

### Maternal care in Finland

Finnish prenatal care is free-of-charge and practically all women use it, indicating that it has good acceptance among the users. Parturients start their prenatal visits on average in the tenth week of gestation, and they have some 14 visits to a special maternity clinic and three visits to a hospital outpatient clinic during pregnancy. Maternity outpatient clinics are part of health care centres which are decentralised into local communities where they are near to those in need of care. One in five women are hospitalised during pregnancy. Practically all women gave birth in public hospitals. The delivery hospital system is centralised on three levels (university, central and local hospitals), but a well-functioning referral system exists for women who are living in catchment areas of local and central delivery hospitals. [42]

EU citizens or everyone with a permanent residency are entitled to (almost) free health and social services. In Finland we know that adults of ethnic minority groups (except refugees) use health care services less than people with Finnish origin. One exception is young migrant-origin women aged 15–29 years, who use more health care services, primarily because of their higher pregnancy and fertility rates [41].

The aim of this study is to analyse the access to and use of maternity care services as well as birth outcomes by ethnic minority women in health care system which main constitutional principles are equality and equity. This kind of information is lacking in Finland, but it is needed in order to improve the maternal health care system to meet better the health needs of ethnic minority parturients. Equality and equity mean that every woman – despite her social or cultural background – has sufficient and good quality care prenatally, during birth and after it for securing maternal and child health.

## Methods

The Finnish Medical Birth Register (MBR) has recorded all births taking place in Finland since 1987, and it is currently administered by the National Institute for Health and Welfare (THL). The register includes all live births and stillbirths of more than 22 weeks of gestation or weighing less than 500 grams. The coverage of the register is complete: during the study period only 0.1% of all births were not reported to the MBR, but the MBR is routinely linked to the Central Population Register (data on live births) and to the Cause-of-Death Register (data on stillbirths and early neonatal deaths), after which it is considered to be complete. Data are checked in the MBR and seemingly incorrect information is sent back to the hospitals for correction. For most variables, the data corresponds well or satisfactorily to information found in hospital records. [43,44]

In our study the MBR data for the years 1999–2001 were linked to information in the general population register through the woman's unique personal identification number available in all Finnish register sources. Since 1970 the Finnish Central Population Register has covered information on all inhabitants who are Finnish citizens or permanent residents of Finland, their background (including country of birth, nationality and language) and their family relations. This data is used as a basis of the Population Statistics, which is continuously compiled by Statistics Finland. [44] The record linkage took place in Statistics Finland, and the researchers received only unidentifiable data. The Ethical Committee at STAKES (past National Research and Development Centre for Welfare and Health) approved the study protocol and the data protection ombudsman was informed about the study, as required by law.

Our study data included 6,532 women of foreign origin (3.9%) with a singleton birth in Finland during 1999–2001 (compared to 158,469 Finnish origin singletons). We did not have information about the ethnicity of women's partners, which beside all other factors may also affect women's utilisation of and access to maternity care.

We defined woman's ethnicity by three items: her country of birth, (her nationality) and her mother tongue and consequently, we formed 15 ethnic minority groups which were:1) Finnish, 2) Nordic, 3) Western, 4) former Eastern Europe, 5) former Soviet Union, Russia, 6) Baltic, 7) Middle Eastern, North African, 8) South Asian (for example India, Pakistan, Bangladesh), 9) Chinese, 10) Iranian, Iraqi, Afghan,11) Southeast Asian (for example Philippines, Thailand, Malaysia excluded Vietnam), 12) Vietnamese, 13) African, 14) Somali and 15) Latin American, Caribbean. This type of classification is effective in identifying persons from the first generation ethnic communities [45] who formed our study subjects.

Some ethnic minority groups may have huge ethnic variation within, but the categorisation is done due to their small number. For example, we grouped together all women of African origin into one ethnic minority group excluding Somalis and North Africans. We were able to study Somali origin parturients separately, since their number was enough for statistical analysis and because they are the largest Muslim and African origin group in Finland. All other Africans included women from other parts of Africa, excluding North African women from Morocco, Tunisia, Libya, Algeria and Egypt who formed their own ethnic group with those coming from Middle East. Furthermore, all women coming from various Eastern European countries have been grouped together, as well as all women from Western countries.

The MBR includes various information about the parturients' reproductive history, care received during pregnancy and childbirth, as well as information about perinatal outcomes. As indicators for the care needed and received during pregnancy we used the mean number of maternity health care centre visits and hospital outpatient clinic visits, and the proportion of parturients with none or 1–2 visits to a maternity centre during pregnancy to show insufficient prenatal care [30]. Consequently, we studied hospitalisation before birth, the mode of delivery, pain relief during vaginal childbirth and other interventions performed during childbirth. We used the following birth health outcomes: prematurity (<37 gestational week), low birth weight (<2,500 grams, LBW), birth weight adjusted for gestational age (SGA small for gestational age according to Finnish sex-specific standards [46]), one minute Apgar score, interventions with newborns and perinatal mortality (stillbirth and deaths before age of 7 days). All these issues were studied in terms of the woman's parity (primiparous and multiparous).

The statistical analysis was conducted using frequency tables and adjusted mean values of each ethnic minority group, and the differences between migrant origin groups and the Finnish origin group were tested using Student's t-test and the test of relative proportions. Since migrant origin parturients' age structure is different than women with Finnish background, we calculated age-and parity-adjusted rates by using the age and parity structures among all women with Finnish background as a standard.

## Results

**Figure 1:**
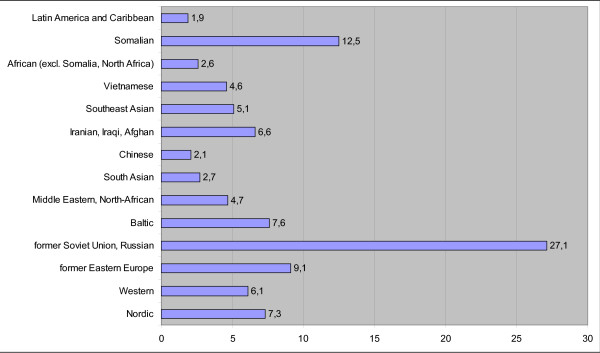
**Singleton births by ethnicity of women in Finland 1999–2001, %**.

The proportion of immigrant women's births increased slightly from 1999 to 2001 (5.4% to 5.9% of all births; 3.8% to 4.0% of singleton births). The largest migrant origin group of parturients were Russians (27.1% of all singleton births of ethnic minority women), Somalis (12.5%) and East-Europeans (9.1%). Proportionally the smallest groups were Latin American and the Caribbean (1.9%) and Chinese (2.1%) (Figure 1).

While one out of five Finnish parturients lived in the most densely populated capital city area, this share was one out of four among migrants. The share was the lowest among women from Iran, Iraq and Afghanistan and women from the Nordic countries (many of them living in the Swedish-speaking areas in the Southern and Western parts of the country). Eighty-three percent of migrants had moved to Finland in the 1990s, i.e. ten years or less before giving birth. This reflects the fact that migration is a relatively new phenomenon in Finland, and our data mainly consists of the first generation migrant women (only 1% of the parturients were second generation).

The mean maternal age among the primiparous was similar among parturients with immigrant and Finnish background (29.9 and 29.6 years, respectively) (Table 1). However, the differences between ethnic minority groups were significant. Mothers from the Nordic (26.8 years) and Baltic countries (28.5 years) were the youngest, while parturients from Southeast Asia and Western countries were the oldest, on average about 31 years old, and parturients from China more than 32 years old. The proportion of parturients aged 35 years or more varied between 7% among Nordic mothers and 29% among Chinese mothers. In general, the proportion of teenage mothers was lower among immigrants than Finns (2.5% vs. 3.0%) with the exception of mothers from Nordic countries (8.0%) and the mothers from the Middle East and North Africa (3.2%).

**Table 1:** Maternal background variables by ethnicity of woman, Finland 1999–2001 (singletons)

		**Age**				**Parity**		**Previous abortions**	**Maternal smoking**
	N	Mean (SD)		-19 years	35+ years	0	4+	abortions	smoking
				%	%	%	%	%	%
Total	165 001	29,9	5,2	3,0	18,3	40,8	4,4	12,7	14,6
Finnish	158 469	29,9	5,2	3,0	18,4	41,0	4,2	12,4	14,8
All migrants	6 532	29,6	5,6 *	2,5 *	18,1	37,6	7,1 ***	19,3 ***	9,2 ***
Nordic	475	26,8	5,4 ***	8,0 ***	7,2 ***	50,2	1,9 ***	16,2 ***	22,7 ***
Western	400	31,7	4,9 ***	1,0 ***	22,5 ***	46,8	1,8 **	9,8 ***	7,5 ***
former Eastern Europe	597	29,1	5,3 ***	1,5 ***	14,7 ***	34,9	5,7 *	10,2 ***	14,1
former Soviet Union, Russian	1 770	30,3	5,9 **	2,4 **	23,2 ***	42,5	1,2 ***	37,2 ***	13,7 **
Baltic	496	28,5	5,5 ***	2,8	2,9 ***	42,2	1,6	30,8 ***	10,5 ***
Middle Eastern, North-African	310	28,9	5,6 ***	3,2	15,8 ***	42,9	5,8	7,4 ***	10,6 ***
South Asian	176	28,9	5,0 **	0,6 ***	13,1 ***	45,7	1,1	7,4 ***	1,7 ***
Chinese	135	32,3	4,7 ***	0,0 ***	28,9 ***	57,5	0,7 **	31,9 ***	0,7 ***
Iranian, Iraqi, Afghan	428	29,3	6,0	2,8	20,1 ***	30,7	4,3 ***	5,6 ***	1,2 ***
Southeast Asian	336	31,0	5,0 ***	0,6 ***	21,7 ***	35,5	1,5 *	12,2	6,3 ***
Vietnamese	302	29,3	5,8	2,0 ***	19,9 **	35,8	7,0	14,9 ***	2,0 ***
African (excl. Somalia, North Africa)	169	30,7	4,9 *	0,6 ***	20,1 ***	25,1	13,8 ***	20,1 ***	1,2 ***
Somalian	817	28,9	5,3 ***	2,6 *	13,1 ***	13,6	30,8 ***	3,3 ***	0,7 ***
Latin America and Caribbean	121	30,8	5,4	1,7 ***	22,3 ***	47,9	0,8	16,5 ***	6,6 ***

From ethnically Finnish 12% of the parturients were single mothers. Among migrant origin groups this share was the highest among Nordic mothers (25%), who were also the youngest ones. Also, among Vietnamese parturients more than every fifth were neither married nor cohabiting. Whereas, most women from other ethnic minority groups were married reflecting the cultural family practises. The share of single mothers was the lowest for mothers from South Asia (5%), the Middle East and North Africa (8%) and Somalia (9%).

The variation in age distribution and fertility patterns between migrant groups explains the large parity differences as shown in Table 1. More than half of Chinese and Nordic parturients gave birth for the first time, while 31% of Somalis and 14% of Iraqi, Iranian and Afghan women and African origin women had four or more previous births. The share to have at least one previous induced abortion was the highest among women originating from countries which traditionally have high abortion rates: the former Soviet Union and Russia (37%), China (32%) and the Baltic countries (31%). Parturients from mainly Muslim countries had induced abortion least of all: the share varied from 3% among women from Somalia to 7% among women from the Middle East and North Africa.

Maternal health habits varied according to woman's ethnicity (Table 1). Women from the Nordic countries (23%) smoked during pregnancy more often than Finnish women (15%). Women from East European countries, the former Soviet Union and Russia had similar smoking rates (14%) than Finns, while all other migrant origin women smoked less often. Only 1–2% of women from Africa (excl. Somalia and North Africa), Iran, Iraq, Afghanistan and China reported maternal smoking.

Ethnic minority women participated substantially in prenatal care. Only 0.2% of migrants had no prenatal care and 0.3% had one or two visits only. Similar figures were found for ethnic Finns (0.3% and 0.2%). Even though some variation between migrant origin groups were observed (from 0% to 0.6% and from 0% to 1.4%, respectively), none of these reached statistical significance. The results were similar when studied by parity (data not shown).

Ethnic minority women had, on average 15.6 prenatal visits to maternity clinic during their pregnancy, 16.1 for primiparous and 15.3 for multiparous women. The ethnic Finnish had on average 1.2–1.5 visits more (P < 0.001), but adjustment for age and parity decreased these differences (data not shown). Women with a Nordic background had almost as many visits as women with Finnish origin, while other migrant groups had less visits. The smallest number of visits was registered for primiparous women from the Middle East and North Africa (mean 15.2 visits, 2.2 less than Finns) and multiparous women from Vietnam (mean 13.8 visits, 2.4 less than Finns).

The difference in hospital outpatient visits was smaller between ethnic minority women (on average 2.7 visits) and women with Finnish background (2.8 visits) (P < 0.001). However, the variation between migrant origin groups was significantly larger: while women from Vietnam had 2.0 visits, women from Africa (excluding Somalia and North Africa) had 3.5 visits during pregnancy. This difference was even larger for multiparous women (1.8 and 3.5 visits, respectively).

In total, 86.4% of women with Finnish origin had at least one ultrasound scan during pregnancy, while the percentage was slightly lower for migrants (85.3%, P < 0.01). Women from Western countries (80.8%) and the former Soviet Union and Russia (82.6%) had less scans (P < 0.001), whereas Vietnamese women (93.7%) had ultrasound scans more often than Finns (P < 0.001). Ethnic minority women had less often amniocentesis (2.9%) or chorionvillusbiopsy (0.4%) than ethnic Finnish women (3.8% and 0.7%, respectively, P < 0.001 and P < 0.01). By migrant origin group, the only statistically significant differences were found for women from the Nordic countries (amniocentesis 1.9%, P < 0.05), South Asia (amniocentesis 0.6%, P < 0.05) and Somalia (amniocentesis 0.5%, P < 0.001 and chorionvillusbiopsy 0.0%, P < 0.05, respectively). Chorionvillusbiopsies were carried out most often on Chinese and Vietnamese women (1.2% both).

During their pregnancy Chinese and Latin American women had pre-eclampsia more often than others (1.2% and 0.7% respectively). Asphyxia was most common among South Asian (6.4%), Chinese (4.2%) and Somali (4.1%) women. Southeast Asian women had a breech or other abnormal presentation more often than other migrant groups (8.6% vs. 4.7%).

Among all the parturients, hospital care due to bleeding, threatening preterm delivery or hypertension before birth was most often recorded for African origin women followed by Finns origin (13.4% and 9.0% respectively). The most common reason for hospitalisation was a risk of preterm birth for Africans (5.7%), for Chinese (6.6%), for Southeast Asians (6.3%) and for Vietnamese (5.2%), while Russian (2.3%) women were most often hospitalised for vaginal bleeding which was also the second most often mentioned reason with Vietnamese women (2.9%).

Among primiparous women, similar amount of Finnish and migrant origin women had an instrumental delivery (forceps or vacuum extractor). Women from the former Soviet Union and Russia (8.8%) and Somalia (8.1%) had less often instrumental deliveries than Finns did (P < 0.01 for both). The differences were smaller for multiparous women, and the difference in instrumental delivery rate between Finns (2.6%) and immigrants (2.2%) was statistically insignificant.

**Figure 2:**
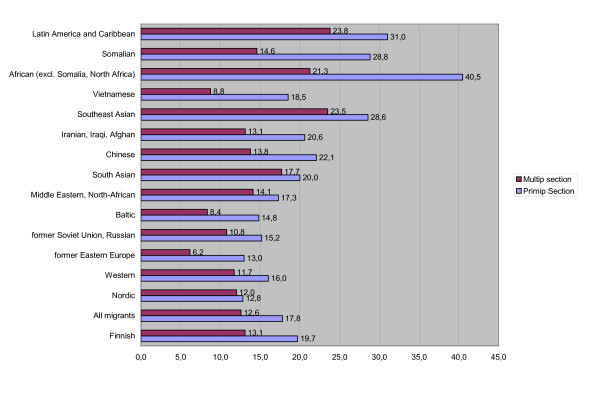
**Caesarean sections by ethnicity of woman in Finland 1999–2001, singleton births, %**.

Primiparous migrant origin parturients also had less often a caesarean section than women with Finnish origin (18.2% vs. 19.7%, P < 0.05, Figure 2). But when studied by ethnicity, women from Africa (excl. Somalia and North Africa) (40.5%, P < 0.001), Latin America and Caribbean (31.0%, P < 0.05), Southeast Asia (28.6%, P < 0.001) and Somalia (28.8%, P < 0.001) had significantly higher section rates. The lowest caesarean section rates were found among women from the Nordic countries (12.8%, P < 0.01), East European countries (13.0%, P < 0.001) and Baltic countries (14.8%, P < 0.05). Among the multiparous parturients, the difference in the section rate was statistically insignificant among Finnish origin and migrant origin women (13.1% and 12.7%, respectively). Women from East European countries (6.2%, P < 0.01) and the Baltic countries (8.4%, P < 0.05) had the lowest section rates than Finns; multiparous women from Southeast Asia (23.5%, P < 0.001) and Latin America and the Caribbean (23.8%, P < 0.05) had the highest section rates.

Including vaginal deliveries only, any pain relief was reported for 89% of primiparous Finns and 90% of primiparous immigrant women. Migrant origin women received epidural analgesia more often than Finns (60% and 40%, P < 0.001). Similar findings were observed for multiparous parturients both for any pain relief (70% and 68%, P < 0.01) and epidural analgesia (23% and 19%, P < 0.001). The only exception was multiparous Vietnamese women, who had lower rates of any pain relief than Finnish origin women (70% and 56%, P < 0.05).

**Figure 3:**
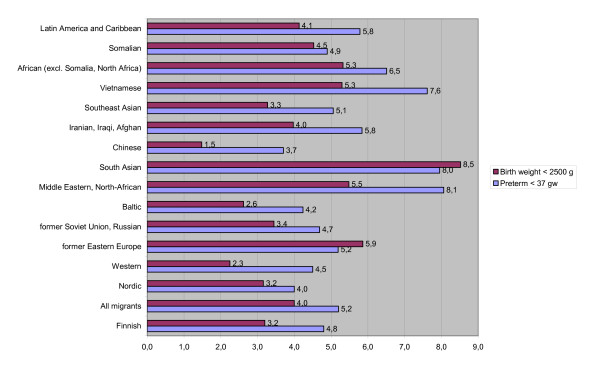
**Preterm and low birth weight newborns by ethnicity of mother, Finland 1999–2001, singleton births, %**.

Migrant origin women gave birth slightly more often prematurely (< 37 gestational weeks) than Finnish-origin mothers (5.1% and 4.8%; Figure 3), but the difference remained insignificant (Figure 3). Mothers from the Middle East and North Africa (8.1%, P < 0.01) and South Asia (8.0%, P < 0.05) had an increased prematurity risk.

Consequently, the newborns of ethnic minority women were more often of low birth weight (< 2,500 grams) than ethnic Finnish (3.8% and 3.3%, P < 0.01, Figure 3). As expected, Asian origin women with smaller body sizes had a higher risk, as the data for South Asians (8.5%, P < 0.001) and Vietnamese (5.3%, P < 0.05) shows. The use of a single cut-off point for being low-birth weight may exaggerates the health risk, since the ethnical variation of body size could not be adjusted in our data. Furthermore, women from East European countries (5.9%, P < 0.001), the Middle East and North Africa (5.5%, P < 0.05) and Somalia (4.5%, P < 0.05) had a statistically significantly increased low birth weight risk.

The risk of being small-for-gestational age (SGA) was similar to low birth weight (Table 2). Newborns of ethnic minority groups had a higher SGA risk than ethnic Finns (2.7% and 2.0%, P < 0.001). The risk was increased for women from South Asia (5.7%, P < 0.001) and Vietnam (4.6%, P < 0.01), Somalia (4.7%, P < 0.001), the Middle East and Northern Africa (4.2%, P < 0.01) as well as among women from Iran, Iraq and Afghanistan (3.5%, P < 0.01).

**Table 2:** SGA and treatment of newborn by ethnicity of mother in Finland 1999–2001, singleton births only, %

		SGA	Treatment to newborn	
			Intensive ward care	Intubation
	N	%	%	%
Finnish	158469	2,1	9,2	0,9
All migrants	6532	2,8 ***	8,2	0,8
Nordic	475	1,5	6,7	0,6
Western	400	1,5	6,5	0,5
former Eastern Europe	597	3,2 *	8,5	0,2
former Soviet Union, Russian	1770	2,0	8,8	0,8
Baltic	496	1,8	6,3	0,2
Middle Eastern, North-African	310	4,2 **	8,4	1,3
South Asian	176	5,7 ***	10,2	0,6
Chinese	135	1,5	5,9	0,0
Iranian, Iraqi, Afghan	428	3,5 *	10	1,2
Southeast Asian	336	3,3	6,5	0,9
Vietnamese	302	4,6 **	5,3	0,0
African (excl. Somalia, North Africa)	169	1,2	8,9	0,6
Somalian	817	4,7 ***	8,9	2,1
Latin America and Caribbean	121	2,5	13,2	1,7

*p < 0.05

**p < 0.01

*** p < 0.001

Migrant origin newborns had more interventions after birth than newborns of Finnish-origin. Intensive care ward was most often needed by the newborns of Latin Americans and Caribbean (13.2%), of South Asians (10.2%) and of Iranians, Iraqis and Afghans (10.0% compared to 5.5% Finns). Respiratory care was given most often to Latin American and Caribbean newborns (1.7%) and to Middle-East and North-African newborns (1.6% compared to 1.0% of Finns). Intubation of newborns was needed most often when mother was from Somalia and Latin America (2.1% and 1.7% respectively compared to 0.6% among Finns). Phototherapy was given more often to Chinese and Vietnamese newborns (11.9% and 9.6% respectively compared to 5.7% of Finns). And lastly, antibiotics were more often given to African, South Asian and Russian origin newborns (4.1%, 4.5% and 4.1% respectively compared to 3.5% of Finns, data not shown).

**Figure 4:**
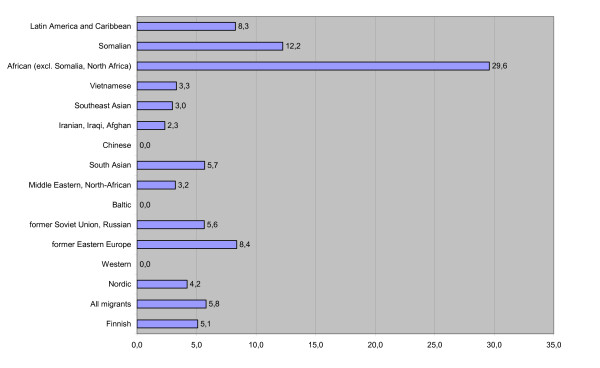
**Perinatal mortality by ethnicity of mother Finland 1999–2001, singleton births, 1/1000**.

The perinatal mortality rate was somewhat, but not statistically significantly, higher for migrant origin newborns (5.7/1000) than for newborns of Finnish origin (5.1/1000) (Figure 4). African women (excl. North Africa and Somalia) had a statistically significantly higher risk for perinatal death (29.6/1000, P < 0.001). The same was also true for women from Somalia (12.2/1000, P < 0.01). Consequently, on average one minute Apgar scores were the lowest for African and Somali origin newborns (8.3 and 8.4 respectively). The highest scores were found among children of Baltic and Western origin (8.8 for both), which was even higher than for Finns (8.6).

## Discussion

Much attention has been given to the "healthy migrant-effect" as an explanation for migrants' positive health outcomes when arriving to their new country of residence, since the youngest and the healthiest have most resources to migrate. Additionally, perinatal outcomes of some foreign-born women having many demographic and socioeconomic risk factors, still have good health outcomes in childbirth which has been termed an epidemiologic paradox. [47] On contrary to these two epidemiological phenomena, our study showed disparities in birth outcomes among migrant origin women compared to Finns.

Our most important and alarming result was the six-fold perinatal mortality rate of African origin singleton newborns and two-fold rate for Somali origin singleton newborns. Due to small number of cases, we were not able to analyse the causes-of-death in more detail [48]. Increased perinatal death rates among ethnic minority groups are not unique in Finland since the similar infant mortality rates have also been reported elsewhere [50] e.g. in the Netherlands [51-53]; in Sweden [32], in Norway [35,54], in the UK [19]; in Ireland [2] and in Italy [37].

In our study the increased prenatal death rates could be associated to the increased risk of low birth weight (LBW) and risk for the small for gestational age (SGA) for Somali origin newborns (LBW 4.5%, P < 0.05; SGA 4.7%, P < 0.001), but not for African origin newborns. Also newborns of South Asian and of Middle East and North Africa origin had increased risks of prematurity and low birth weight which did not end to such fatal outcomes like among African and Somali mothers. Gestational age varies by ethnic background [48,49], but such information is unavailable for the Nordic countries, neither these have been used in clinical practice in Finland. Ethnic-specific perinatal health indicators should be developed and utilised both in clinical practise and in investigating the connection of these different perinatal outcomes in more detail.

Both African and Somali origin primiparous women had the highest caesarean section rates compared to any other group. During their pregnancy African origin parturients had hospital care due to vaginal bleeding or hypertension more often than others. After delivery newborns of African origin were given more antibiotics indicating more infectious diseases in the birth, and during pregnancy. During pregnancy Somali origin newborns were diagnosed to have more asphyxia and Somali origin parturients to have more than others insulin treatment for diabetes. After birth Somali origin newborns had more intubations showing an increased need for special treatment. According to our data multiparous African and Somali origin women had more visits to the hospital outpatient clinics during their pregnancies than any other ethnic group. This indicates that they should have got all needed care and treatment to avoid at least some part of these perinatal deaths. Many of these African but especially Somali origin parturients were multiparous (14% and 31% respectively) and thus they were expected to be familiar with the Finnish maternity care system, knowing its practices and functioning and therefore being able to find help when ever needed, as well as, to be familiar with their pregnancy and childbirth related experiences and health needs.

Single motherhood, infavourable maternal age, and lower socio-economic position are shown to be important risk factors of higher infant mortality [32,50,54]. In our data, African and Somali origin mothers who experienced perinatal mortality were more often unmarried (18.4%) and older (mean 31.7 years) compared to those with surviving newborns in their groups (12.8% and 29.1 years). Our data was too small to study this in more detail, and it did not include variables related to socioeconomic position. In the Netherlands, ethnic infant mortality rate differs also according to generational status and age at immigration of the mother [52] which is related to acculturation and selective migration. Unfortunately, in our data we are not able to study this issue, since we have only information on first generation migrants and we lacked information on woman's age at migration.

Perinatal deaths among ethnic minority population are partly preventable by improving the efficacy of care given during pregnancy and child birth, since suboptimal care factors has been given as one possible explanation for the higher mortality rates [33,53-56]. Studies in Norway [54], in the Netherlands [56], and in Sweden [32] have reported the following care-related problems among migrants: late start of antenatal care, insufficient attendance in antenatal care, a delayed notification by the caregiver about obstetrical problems (e.g. rupturing of membranes, decrease in foetal movements), failure for care givers to act on non-reassuring foetal status or incorrect assessment of labour progression, unidentified or inadequate management of intrauterine growth restriction or decreased foetal movements, inadequate medication, misinterpretation of cardiotomography, and inadequate communication or interpersonal miscommunication with care providers. Also mother's delay in seeking health care and her refusing caesarean sections have been given as a factors related to suboptimal care.

European comparative perinatal death audit study [53] explicitly stated that non-western women constituted a risk group for sub-optimal care factors in infant deaths. This study concluded that suboptimal factors possibly contributed to the fatal outcome in 46% of cases. The most common suboptimal factors were care givers' failure to detect severe intrauterine growth retardation (IUGR 10% of all cases) and smoking in combination with severe IUGR and/or placental abruption (12%). In Finland, however, the suboptimal care factors in perinatal deaths were found to be minor compared to other countries. [53] Care providers' cultural and medical competence when caring for non-western migrant mothers is at the stake. In the long run, ethnic health disparities in maternal, foetal and infant health may be preventable by reducing ethnic inequalities in socio-economic positions. It is also alarming according to one Dutch study [57] that migrant origin women themselves are in a more than three-fold risk of death from maternity related conditions than their Dutch counterparts.

What can western care providers learn from migrant origin women's maternal care experiences? In one Canadian study [58], Somali pregnant women felt that their needs were not always adequately met by care providers, with women reporting unhappiness with both clinical practice and the quality of maternal care they received. In the UK [59], Somali women reported that they were denied information in prenatal care due to punitive attitudes and prejudiced views among health professionals toward them. In the US [60], Somali women considered their childbirth experience positive in general, but they reported racial stereotyping, apprehension of caesarean births, and concern about the competence of medical interpreters as negative aspects of maternal care. Women wanted more information about events in the delivery room, pain medications, prenatal visits, interpreters, and roles of hospital staff. In another US study [61], Somali women were well informed on healthy prenatal practices and compliant in following them. They were generally pleased with the care that they received and accepted most of the diagnostic and therapeutic interventions, but they preferred practitioner who was informed about female circumcision and conservative in the decision to perform section deliveries because many of them are afraid of section [also [3,36]]. In our study African and Somali origin mothers – having the worst birth outcomes- were the visible ethnic minority groups of which Somalis are said to be situated in the lowest level of Finnish ethnic hierarchy according to public opinion [62]. Interview or ethnographic study is needed to investigate specifically migrant origin women's discrimination experiences in maternity care.

The parturient's concept about good maternal care is quite similar all over the world despite woman's ethnicity, i.e. respectful, safe and understandable care. Ethnic minority women do have some special preconditions for a good care depending on her socioeconomic background, on education and on her migration history (reason for migration, from where she has migrated, with whom to which country, at what age and how long time ago). In the US [63] Somali women recognised that a good healthcare practise is characterised by effective verbal and nonverbal communication, feeling valued and understood, availability of female interpreters and clinicians and sensitivity to privacy for gynaecologic concerns. Access to healthcare services and investment in community-based programs to improve women's health literacy were stated to be the prerequisite to good health care system by Somali study subjects. In Australia [64,65] Vietnamese, Turkish and Filipino women appreciated safe, kind, supportive, and respectful care. They expressed less satisfaction with care during labour and birth compared to Australian origin women and communication problems were named to be one reason for being unsatisfied with care. In the same vein, in Denmark [66], Turkish immigrant parturients said that communication problems were a risk to good quality care which often resulted in mutual misunderstandings between care provider and patient. The lack of continuity of the care was an additional strain for these women. The difficulties in communication are potentially dangerous, increasing the risk of delayed care or the risk of missing obstetrical care and intervention. In theory, migrant origin patients in Finland have legal right for official interpreter in medical encounters. There is no information about how much they are used and how well interpretation function for all those involved in medical encounter.

In our study the good news is that there was small variation in the use of and access to inpatient and outpatient prenatal care by woman's ethnicity. One explanation for this is that pregnant woman is required to visit to maternity clinic the first time before 16 gestational weeks in order to get maternity benefits. But still, migrant origin women seem to accept maternity care well since they visited in the clinic almost as frequently as Finns. Thus, the lesser use of maternity care does not explain these ethnic differences in birth outcomes.

But the content of care during labour seem to vary according to mother's ethnicity, since we know from previous studies elsewhere in Europe that there are considerable variations in care procedures given to and needed by migrant origin women during labour. Caesarean section rates are considerable higher for some ethnic minority groups [4,34,36,67]. These findings are in accordance with our study, where the most caesarean sections were performed on primiparous women of African origin (41%), followed by women of Latin American and Caribbean origin (31%) and Somali origin (29%), while only 13% of Nordic and East European origin women had caesarean sections. Only mothers of Latin America had the risk of repeated caesarean section, reflecting the high section rates in their countries of origin. They also had slightly more often pre-eclampsia during their recent birth which may partly explain their higher numbers of sections. In vaginal births Latin American and Caribbean origin women received pain relief more often than others. Additionally their newborns got considerable more often different interventions after birth (intensive ward care, respiratory and intubations) compared to others. To study the cultural meanings of pain in childbirth, qualitative studies are needed to investigate these women's birth and care experiences.

The reproductive health of ethnic minority women is at least three dimensional affected by the woman's social position determined by her ethnicity, class position of her household and her gender both in her country of origin as well as in the new host country [11,19,68-70]. All these categories are hierarchical (and in interaction with each other). Additionally, women of non-Western origin may be at risk of discrimination and deprivation based on gender, class and ethnicity in the Western countries. All these factors can potentially and adversely affect their health and utilization of services. Woman's time of residence in new home country and the level of acculturation may modify the effects of these hierarchies to her health in general and during pregnancy in particular.

We used population-based nationwide register data to study parturients in various ethnic minority groups. This information about woman's ethnicity must not be included in the routine register due to strict ban to register ethnic origin. Therefore, we had to link population register data from another organisation. This caused extra delays and costs for the study. In this register based study we did not have information directly from women's health needs and their realisation during pregnancy, which will affect to the utilisation of care and to the access to care, but we were able to study how the diagnosed risks during pregnancy and birth outcomes are related to given and received care procedures and visits to maternity care among different groups of ethnic minority women.

## Conclusion

Because there are serious differences in birth outcomes and in the care procedures given to and used by ethnic minority women during pregnancy and childbirth, maternity care practises should be re-examined carefully to see whether they systematically vary by mother's ethnicity and by non clinical factors. Empowering of migrant background women and organizing supplement training for public health nurses, midwives and doctors are needed to build together maternity care that is culturally sensitive and respond better to the health needs of different pregnant women and their newborns. Furthermore, to enhance best possible and long lasting health and wellbeing of all mothers and their newborns, some migrant origin women groups are in need of particular type of help and support also after delivery because many of them have less advantageous social position in the West (see for example 71).

## Competing interests

The authors declare that they have no competing interests.

## Authors' contributions

MM and MG planned, participated in the design of the study, performed the statistical analysis and wrote together the manuscript. Both authors read and approved the final manuscript.

## Pre-publication history

The pre-publication history for this paper can be accessed here:


